# Effects of the advanced mandibular spring on mandibular retrognathia treatment: a three-dimensional finite element study

**DOI:** 10.1186/s12903-022-02308-w

**Published:** 2022-07-05

**Authors:** Cheng Zhu, Ruomei Li, Lingjun Yuan, Yikan Zheng, Yu Jin, Hairui Li, Chao Liu, Lunguo Xia, Bing Fang

**Affiliations:** 1 Department of Orthodontics, Shanghai Ninth People’s Hospital, Shanghai Jiao Tong University School of Medicine, Shanghai, 200011 China; 2 Translational Medicine Research Platform of Oral Biomechanics and Artificial Intelligence, Department of Orthodontics, Shanghai Ninth People’s Hospital, Shanghai Jiao Tong University School of Medicine, Shanghai, 200011 China

**Keywords:** Orthodontic Appliances, Clear Aligner, Mandibular Retrognathia, Finite Element Analysis, Temporomandibular Joint

## Abstract

**Background:**

The Advanced Mandibular Spring (AMS) was newly developed as a dentofacial orthopedic appliance in conjunctive use of clear aligners to treat Class II malocclusion with mandibular retrognathia in adolescents. This study aimed to launch a biomechanical assessment and evaluate whether the stress patterns generated by AMS promote mandibular growth.

**Methods:**

A three-dimensional finite element model was constructed using images of CBCT and spiral CT. The model consisted of craniomaxillofacial bones, articular discs, retrodiscal elastic stratum, masticatory muscle, teeth, periodontal ligament, aligner and AMS. Mechanical effects were analyzed in three types of models: mandibular postural position, mandibular advancement with AMS, and mandibular advancement with only muscular force.

**Results:**

The stress generated by AMS was distributed to all teeth and periodontal ligament, pushing mandibular teeth forward and maxillary teeth backward. In the temporomandibular joint area, the pressure in the superior and posterior aspects of the condyle was reduced, which conformed to the stress pattern promoting condylar and mandibular growth. Stress distribution became even in the anterior aspect of the condyle and the articular disc. Significant tensile stress was generated in the posterior aspect of the glenoid fossa, which conformed to the stress pattern stimulating the remodeling of the fossa.

**Conclusions:**

AMS created a favorable biomechanical environment for treating mandibular retrognathia in adolescents.

**Supplementary Information:**

The online version contains supplementary material available at 10.1186/s12903-022-02308-w.

## Background

Skeletal Class II malocclusion is of high prevalence, among which mandibular retrognathia is a frequent characteristic [1]. Adolescents with skeletal Class II malocclusion are treated in two stages. In the first stage, dentofacial orthopedics is carried out with functional appliances, which aims to enhance mandibular growth and potentially avoids tooth extraction or future orthognathic surgery. Functional appliances with pushing rods, such as Herbst, have been proved to effectively correct the sagittal intermaxillary jaw relationship [2] and improve facial profile [3, 4]. Herbst applies constant pushing force on the mandible and shows the highest efficiency among the common functional appliances [5].

With the development of digital design and biocompatible materials, the treatment efficiency of clear aligners has improved in recent years. Clear aligners are favored by teenagers and parents for their minimal influence on eating and reduced number of follow-up visits. However, they can’t achieve orthopedic treatment. In this study, the Advanced Mandibular Spring (AMS) was developed to make up for the deficiency. AMS is a force-adjustable appliance that applies pushing force on the maxilla and mandible via clear aligners to treat Class II malocclusion in adolescents.

In functional appliance treatment, a significant increase in the mandibular effective length is attained by adaptational growth in the mandibular condyle and glenoid fossa remodeling [6]. Condylar chondrocytes have been proved as mechanical sensitive cells [7]. Pressure unloading on the chondrocytes stimulates ossification [8, 9]. The condylar cartilage is the center of greatest growth in the mandible and is associated with morphogenesis of the mandible [10]. Growth of the condyle contributes to increased mandible size and anteroinferior displacement of the mandible [11]. Therefore, the mechanical effect on the condyle and fossa indicates mandibular skeletal adaptation. In addition to skeletal changes, studies found that functional appliances were effective in improving Class II malocclusion due to dentoalveolar changes [4, 12].

Finite element analysis (FEA) has emerged as a useful tool for studying mechanical interaction between tissues. By discretizing the continuous complex structure into numerous nodes and elements, it provides a non-invasive scheme for solving biomechanical problems. Another advantage of FEA is that it studies a homogenous sample while controlling all study variables. The results enable the analysis of stress distribution produced by forces [13]. Changes in the relative level of stress cause a predictable adaptive response in biological tissues [14]. In orthodontic research, FEA has been widely used to study the stress distribution and displacement of teeth and jaws [13]. Duggal’s FEA study on orthopedic treatment suggested that the temporomandibular joint (TMJ) region should be segmented into the condyle, the disc, and the fossa in future research [15], which helps to explore whether the stress pattern was favorable for mandibular growth.

The present study aims to construct a three-dimensional finite element model of oral and maxillofacial hard tissues, articular disc, retrodiscal elastic stratum, muscles, periodontal ligament, clear aligners, and AMS, to study the stress patterns generated by conjunctive use of AMS and aligners, and to explore whether the stress pattern is favorable to mandibular growth and Class II malocclusion treatment.

## Methods

### Design of AMS

**Fig. 1:**
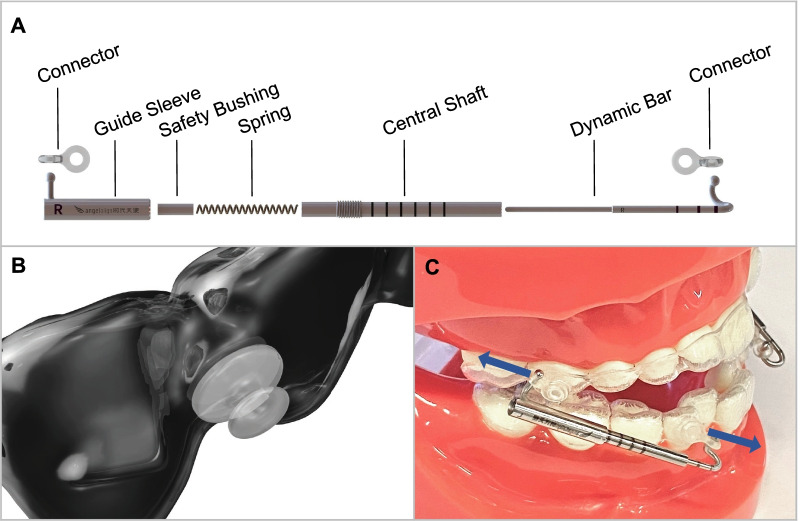
Advanced Mandibular Spring. **A** The design scheme of AMS. The AMS system consists of a nickel-titanium alloy spring, a guide sleeve, a safety bushing, a central shaft, a dynamic bar, and corresponding connectors. **B** Application of buttons with clear aligner. The button is fixed on the clear aligner as an adapter for the orthopedic force. **C** The spring of AMS is compressed to generate pushing force to the mandible and maxilla. The force can be adjusted by screwing the thread on the central shaft, and the six loops on the central shaft correspond to 0–5 N. The force is transmitted through the dynamic bar and guide sleeve, and then applied on aligners using connectors

AMS is designed to apply orthopedic force by generating pushing force on its two ends. The AMS system consists of a nickel-titanium alloy spring, a guide sleeve, a safety bushing, a central shaft, a dynamic bar, and corresponding connectors (Fig. 1A). During clinical application, the mandible is kept at an advanced position with the guidance of AMS. In the advanced position, the spring in AMS is compressed so it generates rebound force along its direction. The connector connects the AMS to the button cemented onto the outer surface of clear aligners (Fig. 1B). The force of the spring is transmitted through the connectors and buttons, and then applied on aligners (Angelalign Inc., Wuxi, China), creating a forward force on the mandibular arch and a backward force on the maxillary arch (Fig. 1C). The force can be adjusted by screwing the thread on the central shaft, and the six loops on the central shaft correspond to 0–5 N. In this work, the force of 5 N of AMS was employed.

### Finite element analysis

#### Case selection

A volunteer with mandibular retrognathia was recruited. Information of the case: female, with Class II division 1, mandibular retrognathia (ANB 6.7°, incisal overjet of 8.5 mm), high angle (MP-FH 39.9°). The volunteer received a spiral CT and CBCT scan. Informed consent was obtained from the volunteer for her participation in the study.

#### Types of the models

AMS’s mechanical effects were analyzed in three types of models, including mandibular postural position (T1), mandibular advancement with AMS (T2), and mandibular advancement with only muscular force (T3). The purpose of including T3 for comparison was that it served as an estimate of physiological stress to verify the safety of AMS as a novel appliance. The position of the mandible and experiment condition are shown in Table 1.

**Table 1:** Types of the models

	T1	T2	T3
Position of mandible	Mandibular postural position	Mandible advanced to Class I molar relationship	Mandible advanced to Class I molar relationship
Condition	without AMS	with AMS	without AMS

#### Geometric model

The geometric model of the dentition and jaw was obtained from the CBCT scan (Promax, 3D Max, Planmeca, Helsinki, Finland) with the following settings: 96 kV, 5.6 mA, exposure time of 13.5 s, slice thickness of 1.2 mm. It was extracted using a deep-learning algorithm provided by Angelalign Inc. Clear borders of bones were identified from the spiral CT scan (SOMATOM Definition Flash, Siemens, Munich, Germany) with the following settings: 120 kV, 350mAs, scan time of 9.61 s, slice thickness of 5 mm, Acq 128*0.6 mm. The model was constructed based on a gray scale threshold between 1250 and 4000. The two models were assembled using best-fitting alignment, and the teeth and alveolar bone from the spiral CT were replaced by those from the CBCT.

The periodontal ligament (PDL) was created by uniformly offsetting 0.3 mm from the surface of the roots of teeth. Boolean operation was done on the alveolar bone to combine the alveolar bone and PDL. Vertical rectangular attachments were designed on upper canines, first premolars and second premolars, and on lower canines and second premolars while horizontal rectangular attachments were placed on upper second molars, lower first molars and second molars. The geometry of aligners was obtained by simulation of the thermo-forming process of aligners. The buttons were positioned on the corresponding aligners of upper first molars and lower first premolars.

The articular disc was modeled based on the anatomical atlas [16] and articular skeletal surfaces. The surface of the articular disc was trimmed and smoothed to match the surfaces of the glenoid fossa and condyle in the model. The retrodiscal elastic stratum was added to the model in mandibular advancement (T2, T3) using oval cylinder following anatomical shape [16].

#### Finite element model

**Fig. 2:**
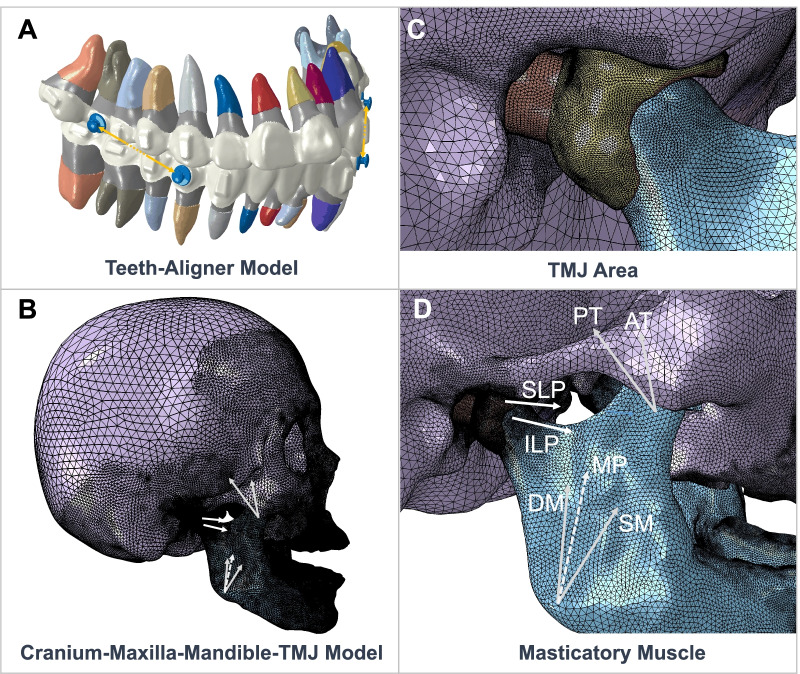
Finite element model. **A** The teeth-aligner model consisted of periodontal ligament, crown and roots of the teeth, attachments, clear aligners, buttons and AMS. **B** The cranium-maxilla-mandible-TMJ model consisted of cranial bones, maxillae, mandible, articular discs, retrodiscal elastic stratum and masticatory muscles. **C** Detailed view of the TMJ area: condyle (blue), articular disc (yellow), retrodiscal elastic stratum (orange) and glenoid fossa (purple). **D** Masticatory muscle consisted of the deep part of masseter (DM), superficial part of masseter (SM), medial pterygoid muscle (MP), superior head of lateral pterygoid muscle (SLP), inferior head of lateral pterygoid muscle (ILP), anterior part of temporalis (AT), posterior part of temporalis (PT)

The finite element analysis was divided into two steps. Firstly, the teeth-aligner model (Fig. 2A) was built to obtain the equivalent force and moment on the alveolar bone with AMS loading. It consisted of PDL, both crowns and roots of teeth, attachments, aligners, buttons and AMS. It’s worth noting that the equivalent force and moment were zero in the model without AMS loading. Secondly, the cranium-maxilla-mandible-TMJ model (Fig. 2B–D) was built to investigate the mechanical effects of AMS on TMJ. It consisted of cranial bones, maxillae, mandible, articular discs, retrodiscal elastic stratum and masticatory muscle. The obtained equivalent force and moment were applied to the alveolar bone.

The geometric models were processed using HyperMesh (Altair Engineering Inc., Michigan, USA). In the teeth-aligner model, teeth and attachments were assumed rigid [17] due to their high relative stiffness to other structures. PDL, aligners and buttons were set as isotropic homogeneous linear elastic materials. The property of PDL was adopted from reported literature [18], while the properties of clear aligners and buttons were provided by Angelalign Inc. Material properties and mesh type are shown in Table 2. 365,916 nodes and 702,968 elements were used in the teeth-aligner model.

**Table 2:** Material properties and mesh type

Component	Young’s modulus (MPa)	Poisson’s ratio	Mesh type
**Teeth-Aligner Model**			
Teeth	rigid	0.30	3D shell triangle
Attachment	rigid	0.30	3D shell triangle
Periodontal ligament	0.47	0.45	3D solid hexahedron
Clear aligner	1000	0.40	3D shell triangle
Button	2000	0.40	3D solid tetrahedron
**Cranium-Maxilla-Mandible-TMJ Model**			
Bone	14,480	0.30	3D solid tetrahedron
Articular disc	16	0.40	3D solid tetrahedron
Retrodiscal elastic stratum	120	0.40	3D solid tetrahedron

In the cranium-maxilla-mandible-TMJ model, the craniomaxillofacial bones, articular discs and retrodiscal elastic stratum were set as isotropic homogeneous linear elastic materials. The properties of bones, articular discs and retrodiscal elastic stratum were adopted from reported literatures [19–21]. The element size of the articular disc, retrodiscal elastic stratum, and elements near alveolar bone and condyle was set 0.3 mm while the element size of rest of the maxillofacial bones was set 2 mm. The element size at the back of the skull was set 5 mm. Material properties and mesh type are shown in Table 2. 561,935 nodes and 2,631,597 elements were used in the TMJ model.

#### Boundary condition

After preprocessing, the finite element model was imported into ABAQUS (Dassault System Inc., Paris, France) to build the boundary condition.

##### The teeth-aligner model

The force generated by AMS was simplified as force vectors and applied to the neck of the button following the designed direction. The contact between clear aligners and teeth was set as hard contact with a friction factor of 0.3. The teeth and PDL shared the node at the roots of teeth. The outside surface of PDL was set as fixed boundary since the stiffness of the alveolar bone was much higher than that of PDL.

##### The cranium-maxilla-mandible-TMJ model

The masticatory muscle activities were simplified as force vectors and spring elements in all types of models. Muscles consisted of superficial and deep part of masseter, anterior and posterior part of temporalis, medial pterygoid muscle, superior head of lateral pterygoid muscle (SLP) and inferior head of lateral pterygoid muscle (ILP). They were applied on the finite element model in sites where the muscles attached to the bones and in the average muscle fiber directions in accordance with the anatomy studies [16].

Muscle forces in maximum clenching have been reported by multiplying Koolstra’s results [22] of the physiologic cross-sectional areas by 0.37 × 10^6^ N·m^−2^ [15, 23, 24]. Considering that the volunteer was high-angle, and the electromyographic (EMG) activity of the masseter and temporalis is stronger in low-angle individuals than in high-angle ones during maximum clenching [25, 26], and the muscle force was linear to EMG signal in isometric contractions [27], the forces in maximum clenching of the masseter and temporalis could be expanded to high-angle cases based on Custodio’s results [25], shown in Additional file 1: Table S1.

Previous studies have shown that the EMG activity of the masseter and temporalis in mandibular postural position was 2–40% of that in maximum clenching [28–30], while SLP showed a higher background activity than the other masticatory muscles [31]. Therefore, in the present study, muscle forces of the masseter, temporalis and ILP in the mandibular postural position were set as 5% of the maximum force, and this ratio was set as 10% for the SLP. The loading of SLP and ILP in different types were set in reference to *Functional Occlusion* [32]: In mandibular postural position (T1), SLP held its contraction to maintain the disc in its correct alignment, and the ILP stayed passive; In mandibular advancement with AMS (T2), both SLP and ILP stayed passive; In the mandibular advancement with only muscular force (T3), the ILP pulled the condyle forward and the SLP released contraction.

To mimic the tensioned retrodiscal elastic stratum in the advancement, a pretention bolt load was applied to it. As the equivalent force was missing in the literature, numerous magnitudes were tried and the minimal magnitude to detach the disc and retrodiscal elastic stratum from condyle was found to be 1.5 N. Loading in different types is shown in Table 3.

**Table 3:** Loading in different types (N)

Component	T1	T2	T3
**Masticatory muscle**			
Masseter (superficial part)	6.75	6.75	6.75
Masseter (deep part)	9.86	9.86	9.86
Temporalis (anterior part)	11.06	11.06	11.06
Temporalis (posterior part)	9.22	9.22	9.22
Medial pterygoid	10.18	10.18	10.18
Lateral pterygoid (superior head)	3.7	0	0
Lateral pterygoid (inferior head)	0	0	4.25
Retrodiscal elastic stratum	0	1.5	1.5
Dentition	0	5	0

The connection between bones and other components was mainly applied using structural coupling. As the retrodiscal elastic stratum and the disc, as well as the disc and the condyle were connected with each other, structural coupling was used following anatomy studies. In consideration of the effect of synovia in the TMJ, frictionless surface-to-surface contacts were established to mimic the movement relation among the condyle, the articular disc, and the glenoid fossa. The nodes around the foramen magnum were rigidly coupled to a restrained reference node in the cervical spine. The reference node was restrained in all degree of freedoms (DOFs) to mimic the support of the skull. The alveolar bone was structural coupling to the corresponding resistance centers of teeth to be applied the reactive forces and moments from the teeth-aligner model. A strong spring element was applied at the chin to mimic the function of digastric muscles. It was added to keep the mandible in balance and avoid unstable deformation. The digastric spring element had a rigidity of 300 N / mm if it transitionally moved and had a rigidity of 2000 N · mm / rad if it rotated.

#### Simulation

The finite element models were simulated using the implicit solver of ABAQUS (Dassault System Inc., Paris, France). The pressure and stress values were in megapascals (MPa) and were interpreted with the color scale (red for the maximum values and blue for the minimum).

## Results

**Fig. 3:**
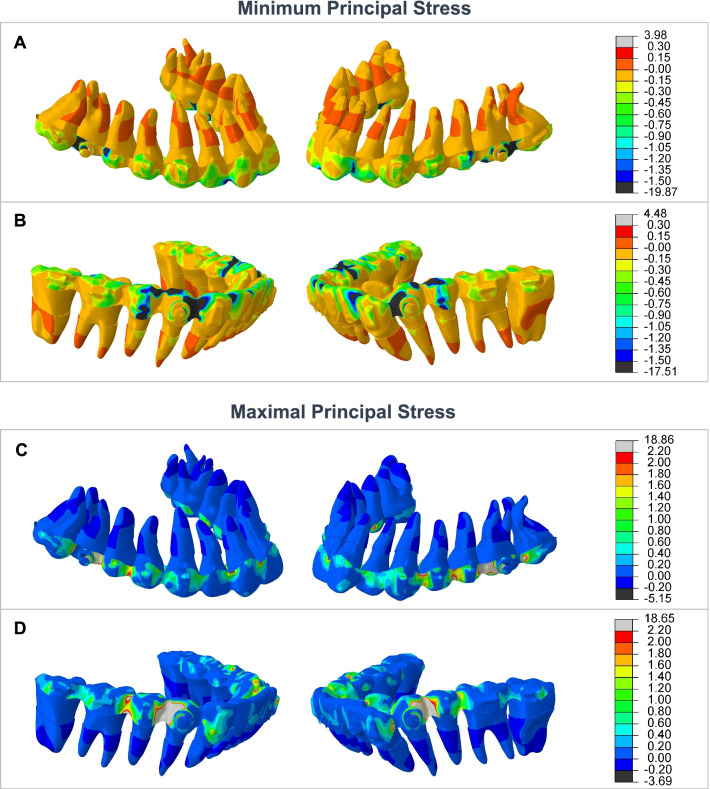
Minimum and maximum principal stress distribution on aligners and PDL in mandibular advancement with AMS. Compressive and tensile stress was distributed on the aligners. The closer to the button, the greater the stress. Compared with the stress on the aligners, lower stress acted on PDL. **A** In the upper dentition, compressive stress mainly acted on the aligner distal to the button. In PDL of anterior(posterior) teeth, slight compressive stress was on the apical 1/2 on the labial(mesial) side and cervical 1/2 on the palatal(distal) side. **B** In the lower dentition, compressive stress mainly acted on the aligner mesial to the button. In PDL of anterior(posterior) teeth, slight compressive stress was on the cervical 1/2 on the labial(mesial) side and apical 1/2 on the lingual(distal) side. **C** In the upper dentition, tensile stress mainly acted on the aligner mesial to the button. In PDL of anterior(posterior) teeth, slight tensile stress was on the cervical 1/2 on the labial(mesial) side and apical 1/2 on the palatal(distal) side. **D** In the lower dentition, tensile stress mainly acted on the aligner distal to the button. In PDL of anterior(posterior) teeth, slight tensile stress was on the apical 1/2 on the labial(mesial) side and cervical 1/2 on the lingual(distal) side

With AMS loaded, stress was distributed on the aligners and PDL (Fig. 3). Negative values of minimum principal stress indicate compressive stress while positive values in maximum principal stress indicate tensile stress. The closer to the button, the greater the stress on the aligners was. The PDL was subject to slighter stress than on the aligners. In the upper anterior teeth, compressive stress was on the labial apical 1/2 and palatal cervical 1/2, while tensile stress was on the labial cervical 1/2 and palatal apical 1/2, making the teeth tip lingually. Similarly, the upper posterior teeth would tip distally, the lower anterior tip labially, and the lower posterior tip mesially. The anteroposterior deformation of dentition showed a backward movement trend of the upper teeth and a forward movement trend of the lower teeth (Additional file 1: Figure S1).

**Fig. 4:**
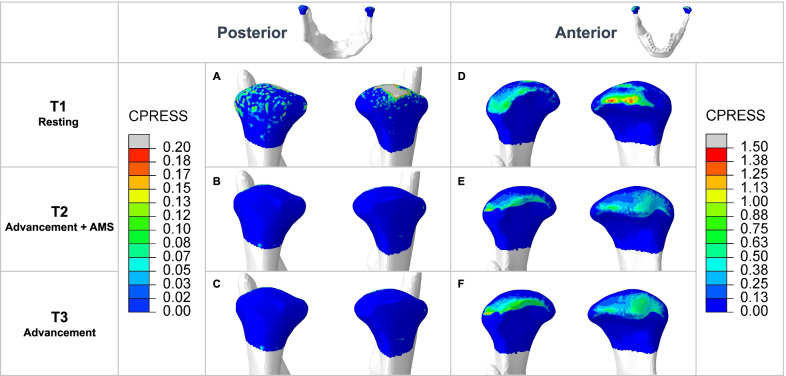
Pressure distribution on the condyle from the posterior and anterior view. **A** In mandibular postural position, the condyles experienced compression in the superior and posterior aspects. **B** In mandibular advancement with AMS, the compression in the superior and posterior aspects was released. **C** In mandibular advancement without AMS, the stress distribution was similar to that with AMS. **D** In mandibular postural position, the condyles experienced compression in the anterior aspect, concentrated on one side of the mediolateral direction. **E** In mandibular advancement with AMS, the compression region moved forward. The stress concentration area in the left condyle disappeared and the pressure was distributed evenly in the mediolateral direction. **F** In mandibular advancement without AMS, the stress distribution was similar to that with AMS

The pressure distribution on the condyle (Fig. 4) showed that in mandibular postural position, the superior aspect and posterior aspect experienced pressure of about 0.1 MPa. With AMS, the pressure was released to near zero. In the anterior aspect, stress distribution became more even in the mediolateral direction. Maximum pressure on the condyle is summarized in Table 4. The anteroposterior deformation of the mandible showed potential backward growth of the condyle (Additional file 1: Figure S2).

**Table 4:** Maximum pressure (MPa) on the condyle

	Posterior		Anterior	
	Left	Right	Right	Left
T1	0.193	0.274	0.950	1.535
T2	0.000	0.000	1.017	0.787
T3	0.000	0.000	1.333	0.755

**Fig. 5:**
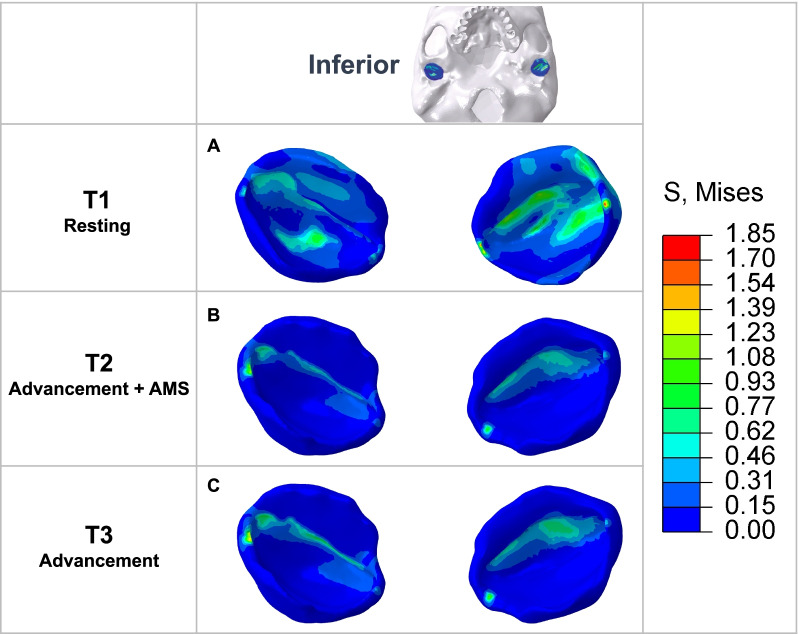
Von Mises stress distribution on the inferior surface of the articular disc. **A** In mandibular postural position, the articular discs experienced stress in the intermediate zone and the anterior band. **B** In mandibular advancement with AMS, the compressive area reduced and moved to the anterior band. The stress distribution tended to be even. **C** In mandibular advancement without AMS, the stress distribution was similar to that with AMS

The results on the disc (Fig. 5) showed that the area subject to compressive stress reduced and the stress distribution tended to be even with AMS loading. Maximum Von Mises stress on the disc is summarized in Table 5.

**Table 5:** Maximum Von Mises stress (MPa) on the articular disc

	Anterior	
	Right	Left
T1	0.613	1.265
T2	0.796	0.605
T3	0.925	0.739

**Fig. 6:**
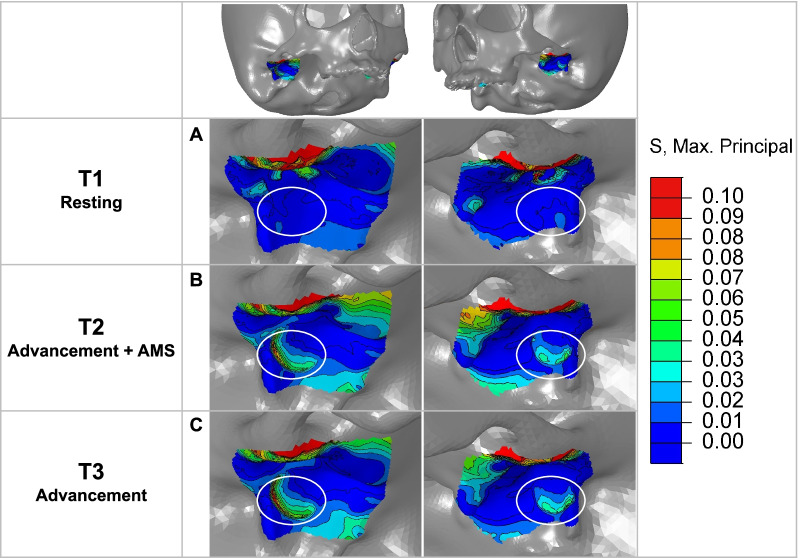
Maximum principal stress distribution on the glenoid fossa. (The white circled area refers to the posterior aspect of the glenoid fossa) **A** In mandibular postural position, the posterior aspect of the glenoid fossa experienced mild tensile stress. **B** In mandibular advancement with AMS, the tensile stress became more significant and extensive. **C** In mandibular advancement without AMS, the stress distribution was similar to that with AMS

In the glenoid fossa (Fig. 6), the posterior aspect of the fossa experienced mild tensile stress in mandibular postural position. With AMS, tensile stress of 0.05–0.1 MPa was generated in the posterior aspect of the glenoid fossa. The maximum of maximum principal stress on the fossa is summarized in Table 6.

**Table 6:** Maximum of maximum principal stress (MPa) in the posterior aspect of the glenoid fossa

	Anterior	
	Right	Left
T1	0.003	0.004
T2	0.091	0.059
T3	0.099	0.072

In T3, the mandible was advanced with only muscular force. The stress distribution with muscular force was similar to that with AMS in the TMJ area, including the condyle, the disc and the fossa. AMS didn’t cause additional burden to tissues.

## Discussion

Functional appliances have been used for over a century to treat Class II malocclusion [33]. They normally keep the mandible in a forward position and activate protractor muscles, which stimulates mandibular growth [2, 34, 35]. They are found to promote upward and backward growth of the condyle and corresponding adaptation of the glenoid fossa in clinical use [36–38].

Scholars have launched FEA on functional appliances to explore their treatment mechanism. Duggal’s model consisted of the maxillofacial bones, the articular disc, articular ligaments, teeth, PDL, alveolar bone, muscles, and miniplate anchored Herbst [15]. Complete components were also included in our modeling process. The geometric model was constructed using images of CBCT and spiral CT. CBCT has been widely used for modelling [15, 24, 39] for its high spatial resolution. With the data from CBCT, we obtained a fine model of dentition and jaw which distinguished roots from the alveolar bone. However, the border of bones was unclear due to its lower contrast resolution and greater image artifacts compared with spiral CT [40, 41]. As a supplement, spiral CT was used. Even if the volunteer gave informed consent, she bore risks of additional radiation exposure. Therefore, the FEA could be conducted in research to explore the biomechanical effects of an appliance, but it shouldn’t be applied to individual patients in clinical practice. The geometric model was then processed with fine mesh in the areas of interest such as the TMJ region to improve precision. Masticatory muscle forces in different jaw positions were adopted to better simulate clinical situations.

Condylar chondrocytes are mechanical sensitive cells [7]. Studies have shown that when condylar chondrocytes experienced pressure unloading as the posterior part of the condyle deviated from the glenoid fossa, they responded biologically to unloading, enhancing their differentiation and maturation, eventually resulting in increased ossification [8, 9] and substantial new bone formation in the posterior aspect of condyle [42]. Existing FEA attributed condylar growth to the increased tensile stress on the condyle in the mandibular advanced position [39, 43], while the results of the present study showed that the initial pressure in the superior and posterior aspects of the condyle was released to near zero by AMS (Fig. 4). A pressure difference of 0.1 MPa in this study would activate condylar chondrocytes and stimulate condylar growth along the unloading direction, upward and backward in this case. The condylar cartilage is the center of greatest growth in the mandible [10]. Growth of the condyle contributes to increased mandible size and anteroinferior displacement of the mandible [11]. The anteroposterior deformation of the mandible also indicated the potential backward growth of the condyle and consequent mandibular anterior displacement trend (Additional file 1: Fig. S2). The result was consistent with Whetten and Johnston’s proposal in the ratchet hypothesis that the condyle could resist episodic compression and grow when unloaded [44]. This study revealed that AMS induced pressure unloading on the condyle, helped create a favorable growth environment for the chondrocytes, therefore for mandibular anterior displacement.

In the anterior aspect of the condyle, the results showed that pressure distribution tended to be even with AMS (Fig. 4). It corresponded to Shrivastava’s [39] FEA results that compressive stress in the anterior aspect reduced after advancement. Studies have shown that continuous compression on the condylar cartilage decreased the proliferation of chondrocytes and the amount of extracellular matrix [45], and induced cartilage thinning [46]. AMS avoided adverse effects in the anterior aspect of the condyle.

On the articular disc, Wu’s study has shown that sustained mechanical loading could significantly reduce nutrient levels of the disc in which cells may die [47]. In the present study, the stress was in the intermediate zone and anterior band in mandibular postural position and was reduced to only the anterior band with AMS (Fig. 5). Shrivastava et al. found that stress in the middle aspect of the disc reduced in mandibular advancement, but that in the posterior aspect increased [39]. We didn’t find stress change in the posterior aspect but identified similar reducing stress in the intermediate zone.

In the glenoid fossa, studies have confirmed that tensile strain and stress promoted the osteogenic differentiation of mesenchymal cells [48]. With tensile stress, mesenchymal cells in the posterior region of the fossa oriented in the direction of the pull of the posterior fiber, migrated or condensed [49], which eventually led to a considerable increase in bone formation in the posterior and middle region of glenoid fossa [50]. In this work, the tensile stress became more significant and extensive with AMS (Fig. 6). A stress difference of 0.05–0.1 MPa could be generated in the posterior aspect to stimulate new bone formation. This could be correlated with the results of Shrivastava et al. [39] where tensile stress was found in the superior and posterior aspects of the fossa in advancement. The results of this study suggested that the use of AMS induced remodeling of the glenoid fossa.

In mandibular advancement with AMS, the stress was distributed on the aligners and then acted on the crowns of all teeth. Previous finite element studies of fixed functional appliances demonstrated similar results that the whole dentition experienced stress but the teeth on which the appliance directly applied force showed the highest stress, mandibular premolars and maxillary posterior in Panigrahi’s study [51] and mandibular canines in Chaudhry’s study [24]. In this study, stress concentrated on the maxillary first molars and mandibular premolars, where AMS was connected to the clear aligners.

The present study showed that the PDL was subject to evenly distributed stress, which was much lower than the stress in the crowns of teeth. In PDL of maxillary anterior teeth, slight compressive stress was on the apical 1/2 on the labial side and cervical 1/2 on the palatal side. In PDL of mandibular anterior teeth, slight compressive stress was on the cervical 1/2 on the labial side and apical 1/2 on the lingual side. This was in accord with previous research that functional appliances like Herbst led to maxillary incisor inclination and mandibular incisor proclination [35, 52], regarded as the dental compensation in the treatment of Class II malocclusion. It could be minimized through different designs in functional therapy [34, 53]. In this study, the anteroposterior movement trend was indicated in the deformation of dentition (Additional file 1: Figure S1). Together with the reconstruction of alveolar bone, it led to an overall backward movement of maxillary teeth and forward movement of mandibular teeth which helped to correct Class II malocclusion.

The three-dimensional mathematical modeling of several components and the boundary setting may have influenced the results of this study. The results on deformation reveal the moving trend rather than the accurate amount. To overcome the limitations, individualized and viscoelastic modeling will be needed to simulate clinical situations and to achieve feedback on detailed structures. This could provide a better understanding of stress changes in craniofacial structures and guide future clinical applications.

## Conclusion

The present study showed that AMS distributed the stress to all teeth with slight and even stress in the periodontal ligament. It would push mandibular teeth and periodontal ligament forward and maxillary teeth and periodontal ligament backward. Mandibular advancement with AMS resulted in stress changes in the temporomandibular joint, including unloading the posterior and superior aspect of condyle, making the anterior aspect of condyle and the articular disc evenly stressed, and yielding tensile stress on the glenoid fossa. AMS created a favorable biomechanical environment for mandibular growth in adolescents with mandibular retrognathia.

## Supplementary Information

**Additional file 1:** Muscle forces in maximum clenching of different vertical facial patterns (Table S1) and anteroposterior deformation of the dentition and mandible (Fig S1–2).

## Data Availability

The datasets generated during and/or analyzed during the current study are available in the Figshare repository, https://doi.org/10.6084/m9.figshare.19336436.v1.

## Abbreviations

AMS: Advanced Mandibular Spring
FEA: Finite element analysis
TMJ: Temporomandibular joint
PDL: Periodontal ligament
