# Supplementary material for: Effects of the advanced mandibular spring on mandibular retrognathia treatment: a three-dimensional finite element study
Source: BMC Oral Health. 2022 Jul 5;22:271. doi: 10.1186/s12903-022-02308-w (PMC9254520; doi:10.1186/s12903-022-02308-w)
Supplement: Supplementary file 1 — Additional file 1: Muscle forces in maximum clenching of different vertical facial patterns (Table S1) and anteroposterior deformation of the dentition and mandible (Fig S1–2). [file 12903_2022_2308_MOESM1_ESM.docx]

**Effects of the Advanced Mandibular Spring on Mandibular Retrognathia Treatment: A Three-dimensional Finite Element Study**

Supplementary Table 1. Muscle forces in maximum clenching of different vertical facial patterns (N)

| Muscle | Low-angle | Normal-angle | High-angle |
| --- | --- | --- | --- |
| Masseter (superficial part) | 136.8 | 135.4 | 135 |
| Masseter (deep part) | 199.7 | 197.6 | 197.1 |
| Temporalis (anterior part) | 222.3 | 222 | 221.2 |
| Temporalis (posterior part) | 185.2 | 185 | 183.2 |
| Medial pterygoid | 203.5 | 203.5 | 203.5 |
| Lateral pterygoid (superior head) | 37.0 | 37.0 | 37.0 |
| Lateral pterygoid (inferior head) | 85.0 | 85.0 | 85.0 |

The deformation values were in millimeters (mm) and were interpreted with the color scale (The value is positive if the movement is backward and negative if the movement is forward).


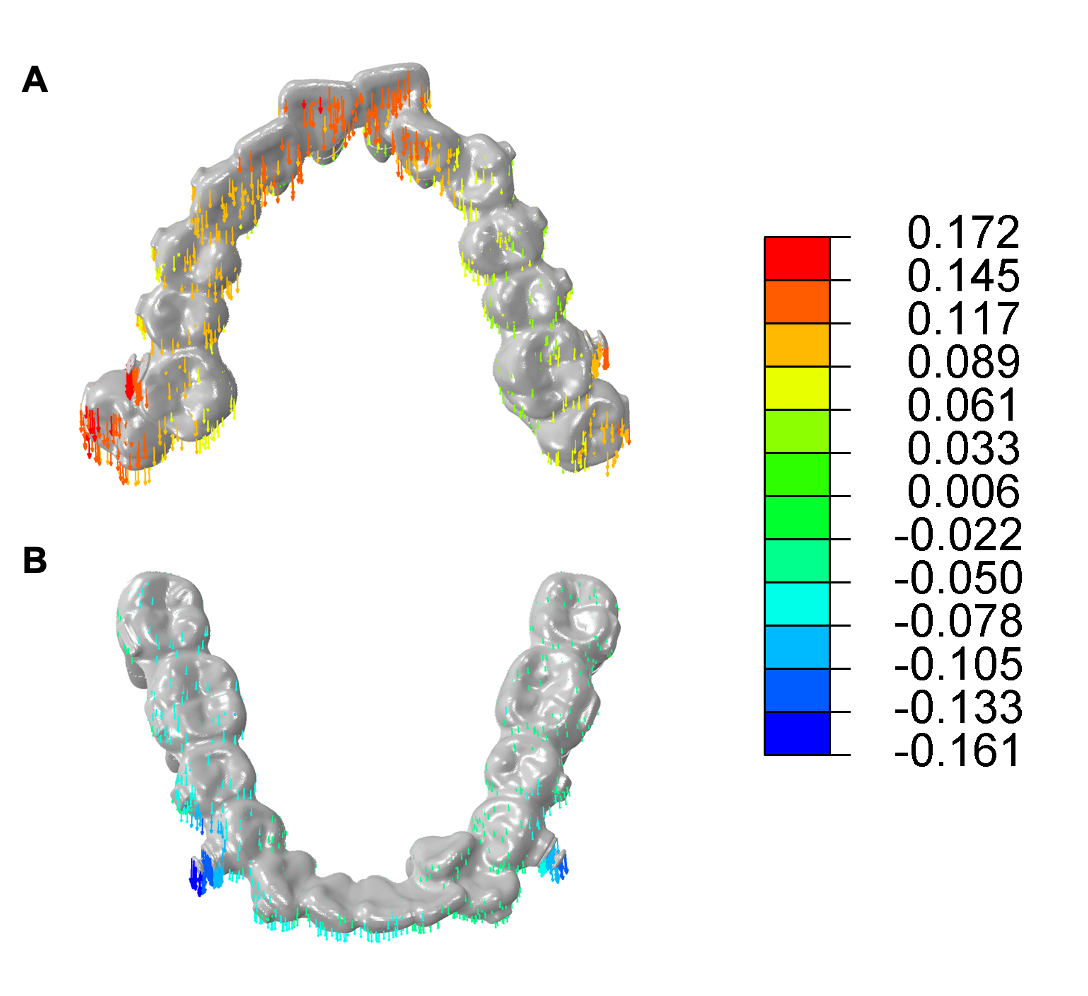


**Supplementary Figure 1. Anteroposterior deformation of the dentition.** The trend of teeth movement is backward in upper teeth and forward in lower teeth.


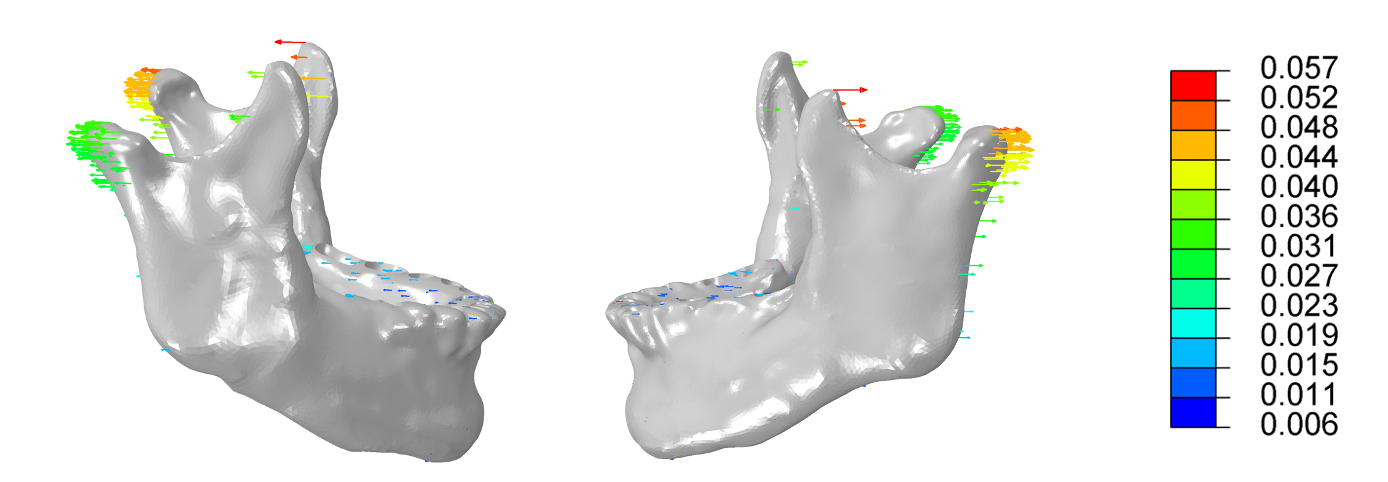


**Supplementary Figure 2. Anteroposterior deformation of the mandible.** The trend of condylar growth is backward in the anteroposterior direction.
